# VEGFR-3 signaling in macrophages: friend or foe in disease?

**DOI:** 10.3389/fimmu.2024.1349500

**Published:** 2024-02-22

**Authors:** Saranya Kannan, Joseph M. Rutkowski

**Affiliations:** Department of Medical Physiology, Texas A&M University School of Medicine, Bryan, TX, United States

**Keywords:** monocyte, M1 macrophage, M2 macrophage, FLT4/VEGFR3, VEGFC/D-VEGFR3/NRP2 axis, lymphangiogenesis

## Abstract

Lymphatic vessels have been increasingly appreciated in the context of immunology not only as passive conduits for immune and cancer cell transport but also as key in local tissue immunomodulation. Targeting lymphatic vessel growth and potential immune regulation often takes advantage of vascular endothelial growth factor receptor-3 (VEGFR-3) signaling to manipulate lymphatic biology. A receptor tyrosine kinase, VEGFR-3, is highly expressed on lymphatic endothelial cells, and its signaling is key in lymphatic growth, development, and survival and, as a result, often considered to be “lymphatic-specific” in adults. A subset of immune cells, notably of the monocyte-derived lineage, have been identified to express VEGFR-3 in tissues from the lung to the gut and in conditions as varied as cancer and chronic kidney disease. These VEGFR-3+ macrophages are highly chemotactic toward the VEGFR-3 ligands VEGF-C and VEGF-D. VEGFR-3 signaling has also been implicated in dictating the plasticity of these cells from pro-inflammatory to anti-inflammatory phenotypes. Conversely, expression may potentially be transient during monocyte differentiation with unknown effects. Macrophages play critically important and varied roles in the onset and resolution of inflammation, tissue remodeling, and vasculogenesis: targeting lymphatic vessel growth and immunomodulation by manipulating VEGFR-3 signaling may thus impact macrophage biology and their impact on disease pathogenesis. This mini review highlights the studies and pathologies in which VEGFR-3+ macrophages have been specifically identified, as well as the activity and polarization changes that macrophage VEGFR-3 signaling may elicit, and affords some conclusions as to the importance of macrophage VEGFR-3 signaling in disease.

## Introduction

Lymphatic vessels preserve tissue homeostasis in health and actively participate in the propagation and resolution of inflammation in disease. If viewed as merely the drainage system of peripheral tissues, lymphatic vessels transport interstitial fluid away from the tissue space. Lymphatic transport is thus the route of macromolecular clearance, e.g., cytokines or antigen, to the draining lymph node. It is also the route of antigen-presenting cells (APCs) to reach the node and the network is often co-opted by metastatic cancer cells. This drainage aspect alone makes lymphatics a critical play in the immune response and cancer dissemination, but lymphatics have become increasingly appreciated for their active roles in regulating immunity. Lymphatic endothelial cells (LECs) interact with APCs to direct their maturation and migration, regulate T-cell populations, and can directly process and present antigens (1, 2). This makes targeting lymphatic vessel biology, or inducing or inhibiting lymphatic network expansion (lymphangiogenesis), an exciting target in regulating cancer, inflammation, and immunomodulation. The predominant signaling pathway that controls lymphangiogenesis is activation of vascular endothelial growth factor receptor-3 (VEGFR-3) by its ligands VEGF-C and VEGF-D. VEGFR-3 activity is not unique to lymphatic endothelium, however, and its expression and important roles in a subset of macrophages is the discussion topic of this mini review.

## VEGFR-3 signaling and its effects

VEGFR-3, also known as Fms-like tyrosine kinase receptor 4 (FLT-4) and encoded by the gene *FLT4/Flt4*, plays several critical roles in LEC function (3). VEGF-C and VEGF-D induce lymphangiogenesis via VEGFR-3; this process constitutes several steps such as cell proliferation, migration, and organization, all regulated by VEGFR-3 (4, 5). VEGFR-3 signaling also impacts cell survival, calcium flux, vessel permeability, and collecting lymphatic vessel contractility (6–8). VEGFR-3 is expressed on the cell surface and can signal through the formation of VEGFR-3/VEGFR-2 heterodimers or VEGFR-3 homodimers. Signaling through VEGFR-3 homodimers predominantly activates ERK1/2 pathways, whereas the PI3K/AKT pathway is induced by VEGFR-3/VEGFR-2 heterodimers (9). Functionally both pathways play an important role in regulating LEC migration (10). The ligands VEGF-C/D also interact with the independent receptor/co-receptor neuroprilin-2 (NRP-2) that associates with VEGFR-3 and activates a VEGF-C/D-NRP-2 signaling axis that controls LEC proliferation, reconstruction, and lymphangiogenesis (11). VEGF-C knockout mice demonstrate failure in lymphatic vessel sprouting, resulting in death due to excessive fluid accumulation in the tissues; a similar phenotype is observed with VEGFR-3 deletion (12, 13). NRP2 knockout mice demonstrate abnormal lymphatic patterning, particularly at the level of lymphatic capillaries (14). Similarly, transgenic mice expressing a soluble form of VEGFR-3 from the skin demonstrated an inhibition of VEGF-C/D signaling that limits dermal lymphangiogenesis and induces regression of already formed lymphatic vessels (15). Patients with hereditary lymphedema can have missense mutations in the VEGFR-3 gene that involves an inactivation of VEGFR-3 tyrosine kinase and more than a third of familial lymphedemas can be explained by mutations identified in the VEGFR-3 signaling pathway (16). While VEGFR-3 is often considered to be restricted to LECs in the adult, during development, it is expressed in both blood and LECs (17, 18). There is also evidence of VEGFR-3 signaling playing critical roles in neural development andinin in the adult brain and neurons (19–21). Several studies have also documented both the expression of VEGFR-3 on macrophages and direct macrophage responses to its signaling.

## VEGFR-3+ macrophages

As an immune cell type involved in the inflammatory response, tissue remodeling, and vasculogenesis, macrophage manipulation could be concerning as an off-target effect should VEGFR-3 be targeted in disease. VEGFR-2-expressing macrophages have been considered and studied in the context of VEGFR-2/VEGF-A inhibition (22–26), but the role and impact of VEGFR-3-expressing macrophages are much less appreciated. This review of the identification and impact of VEGFR-3 signaling in macrophages seeks to highlight this regulatory axis and its potential importance in inflammation.

VEGFR-3+ (R3+) macrophages and their specific activity have been discovered and/or implicated in various pathologies such as sepsis, infection, injury, obesity, fibrosis, chronic inflammatory disease, and cancer (27–32). Several studies have also reported the presence of R3+ macrophages in tissue culture and macrophage cell lines. These findings are summarized in **Table 1** and discussed more thoroughly here. It is important to note that we report only those studies wherein VEGFR-3 is specifically identified, targeted, or manipulated. Likewise, NRP-2+ macrophages have also been identified in several contexts, but it is not possible to discern in these studies if NRP2-VEGFR-3 interactions impact macrophage biology due to this axis not being specifically tested. They may be of interest for further reading (53–57).

**Table 1:** Tissues in which R3+ macrophages have been identified.

Tissue type	Comments	Detection Method	Ref.
Skin	Expressed in dermal macrophages surrounding VEGF-C transfected melanomas in mouse	Immunofluorescence	(33)
Peritoneal exudate macrophages	Expressed on cultured mouse peritoneal macrophages	Flow cytometry	(33)
Cornea	Expressed by the macrophages in posterior stroma of the murine cornea	Immunofluorescence	(34)
Conjunctiva	Expressed by the macrophages in the murine conjunctiva	Immunofluorescence	(35)
Cervix	Detected in human peritumoral macrophages	Immunofluorescence	(36)
Peritoneal exudate macrophages	Expressed in thioglycolate (TG) stimulated peritoneal macrophages in culture	RT-PCR	(37)
Brain	Expressed in brain macrophages following focal cerebral ischemia in mouse	Immunofluorescence	(38)
Urinary bladder	Detected in TAM in murine bladder cancer	Immunofluorescence	(39)
Brain	Enhanced expression in brain post-neurotropic viral infection	FACS	(40)
Brain	Expressed in the murine hippocampal macrophages post epilepsy	Immunofluorescence	(41)
Peritoneal exudate macrophages	Glucose treatment downregulates the expression in the cultured murine peritoneal exudate macrophages	qRT-PCR	(42)
Bone marrow-derived macrophages	Detected in bone marrow-derived macrophages in culture	qRT-PCR	(43)
Peritoneal exudate macrophages	Detected in activated peritoneal macrophages *in vivo* and in RAW264.7 macrophages *in vitro*	qRT-PCR, FACS	(44)
Blood	Expressed by monocytes freshly purified from human blood	IHC, RT-PCR	(45)
Bone marrow-derived mononuclear cells	Expressed on culture CD11b^+^ bone marrow-derived cells that integrated into lymphatic vessel after reintroduction into mice	FACS, IHC	(46)
Peritoneal exudate macrophages	Increased expression following endotoxin shock	qRT-PCR	(28)
Bone marrow-derived macrophages	Higher expression in M1 macrophages	qRT-PCR	(47)
Breast	Expressed by TAM in mouse breast tumor	Flow cytometry, immunofluorescence	(48, 49)
Adipose tissue	Expressed by the macrophages in the murine adipose tissue	qRT-PCR	(50, 51)
Liver	Expressed in the macrophages of murine liver following hepatic reperfusion injury	Immunofluorescence, flow cytometry	(29)
Heart	Expressed by cardiac macrophages in mouse	qRT-PCR	(52)
Intestine	Expressed by colon macrophages in colitic mice	Immunofluorescence, flow cytometry	(27)
Carotid artery	Expressed in the macrophages within inflammatory perivascular regions of human atherosclerotic plaque	Immunofluorescence	(31)

More detailed information as to the cell type described, method used to validate VEGFR-3 expression, and referenced literature is provided.

## R3+ macrophages in disease: friends?

### VEGFR-3+ macrophages signaling in bacterial and viral infection

Ma et al. demonstrated that R3+ macrophages are involved in the active elimination of *Salmonella typhimurium* via AMPK-mediated glycolytic reprogramming and inflammasome activation. Upon infection, macrophages expressed FLT4/VEGFR-3 and its ligand VEGF-C, and this signaling was involved in the inhibition of CASP1 (caspase 1)-dependent inflammasome activation and pyroptosis; it also enhanced MAP1LC3/LC3 activation for bacterial elimination (58). Zhang and colleagues utilized macrophage-specific VEGFR-3 knockout mice (*Vegfr-3* ^fl/fl^LyzM-Cre^+/−^) to demonstrate that activated R3+ macrophage prevents endotoxin shock by limiting Toll-like receptor-4 (TLR-4)/NF-κB signaling and thereby represented a self-control mechanism during antibacterial innate immunity. Gram-negative bacterial infection activated VEGF-C/VEGFR-3 signaling in macrophages and enhanced SOCS1 (suppressor of cytokine signaling 1) expression to inhibit the TLR-4/NF-κB pathway (28). *Vegfr-3* ^fL/fl^LyzM-Cre^+/−^ mice were also used to demonstrate that R3+ macrophages restrain neuronal damage post-encephalitis by limiting TNF-α production. Brain macrophages from *Vegfr-3* ^fl/fl^LyzM-Cre^+/−^ mice infected with Japanese encephalitis virus (JEV) were sequenced to identify some of the top activated signaling pathways that were associated with inflammation and infection including the PI3K-AKT pathway. Peritoneal exudate macrophages (PEMS) from these mice showed reduced levels of p-AKT1 after treatment with JEV-infected N2a (Neuro2a cells). VEGF-C treatment had an inhibitory effect on TNF-α production in wild-type PEMS that was restored by uprosertib, an AKT inhibitor. This suggests that macrophage VEGFR-3 signaling and the activation of the PI3K-AKT pathway inhibits TNF-α secretion in the brain after neurotropic viral infection (40). Taken together, R3+ macrophages appear to exhibit a positive response to infections.

### VEGFR-3+ macrophages signaling in inflammatory bowel disease

In a model of experimental colitis, mice injected with carboxylated crimson, fluorescent LPS-coated beads showed increased clearance of bead+ macrophages from the inflamed colon with VEGF-C treatment (27). Macrophages isolated from colitic mice showed a significant increase in phosphorylated STAT6 upon VEGF-C treatment with the effect being inhibited by an anti-VEGFR-3 antibody. Inhibition of STAT6 in VEGF-C-treated cells abolished the ability of VEGF-C to alter “M2” and “M1” macrophage-associated genes as well as macrophage migration. *In vitro*, VEGF-C-induced bone marrow-derived macrophages ameliorated clinical parameters in the experimental colitic mice, whereas this protective role was abolished by STAT6-silenced VEGF-C-induced macrophages. This suggests that VEGF-C/VEGFR-3 signaling in macrophages provided protection against the development of acute and chronic colitis by increasing cell mobilization and bacterial antigen clearance from the inflamed colon to draining lymph nodes (27).

### VEGFR-3+ macrophages signaling in lung injury

R3+ macrophages contributed to prolonged survival and decreased the degree of lung injury in a sepsis model through reducing TLR-4 signaling and pro-inflammatory cytokines (28). A study by Yamashita et al. (59) demonstrated that R3+ macrophages ameliorated experimental lung injury through a series of gain and loss of VEGFC/VEGFR-3 signaling function experiments that employed adenovirus-mediated intranasal delivery of VEGF-C (Ad-VEGF-C vector) and soluble VEGFR-3 or anti-VEGFR-3 blocking antibodies. They also utilized LysM-specific VEGFR-3 knockout mice produced from LysM-Cre and VEGFR-3-floxed mice and reported that VEGF-C/VEGFR-3 signaling increased efferocytosis via upregulation of integrin α_v_ in macrophages. In addition, they showed a significant decrease in VEGFR-3 expression and efferocytosis in human monocyte-derived macrophages upon incubation with bronchoalveolar lavage fluid from acute respiratory distress syndrome (ARDS) patients, suggesting that the protective function of macrophages is potentially impaired in patients during ARDS due to decreased levels of R3+ macrophages.

### VEGFR-3+ macrophages in wound healing and inflammation

Maruyama and colleagues identified that high-glucose conditions caused a reduction in VEGFR-3 expression in macrophages that could be rescued by stimulation with IL-1β. Following rescue, application of these R3+ macrophages to wounds in diabetic mice induced lymphangiogenesis and accelerated wound healing. The authors posited that these macrophages play a direct role in vessel formation. This suggests that the decrease in R3+ macrophages in diabetic mice may have contributed to a reduction in lymphatic vessels resulting in impaired dermal wound healing (42).

Dermal immunology studies carried out in mice expressing soluble VEGFR-3 (a VEGF-C/D trap) in the skin identified fewer macrophages in the dermal-draining lymph nodes compared to normal mice (60). These mice demonstrated reduced immune tolerization, even developing an autoimmune response to their skin. Increased levels of VEGF-C/D are associated with inflammation and cancer progression and play a critical role in lymphatic and lymph node structure remodeling (61–63). Whether macrophage VEGFR-3 signaling could also play a role in immune response propagation through lymph node sinus remodeling or lymphatic–immune interactions specifically in this mutant model or generally in other models remains unknown. For example, subcapsular sinus macrophages in lymph nodes prevent the systemic spread of lymph-borne pathogens and initiate humoral responses (64). Inhibition of VEGFR-3 signaling reduced not only lymph node lymphatic proliferation and inflammation, but also the expression of IL-1β in subcapsular sinus macrophages following stroke (65). What role macrophage VEGFR-3 signaling plays in lymph node lymphangiogenesis or how VEGFR-3-mediated remodeling impacts polarization during inflammation thus remains unclear.

### VEGFR-3+ macrophages signaling in hepatic injury

A study utilizing a rat model of orthotopic liver transplantation demonstrated a protective role of VEGFC/VEGFR-3 signaling on ischemic reperfusion injury by modifying macrophage polarization from the so-called pro-inflammatory “M1” to beneficial “M2” phenotype. Continuous perfusion of donor livers with VEGF-C prior to transplantation significantly reduced post-operative serum alanine transaminase (ALT) and total bilirubin (TBIL) level and also suppressed the NF-κB activity and upregulated the expression of SOCS1 and p-GSK3β in Kupffer cells and shifted the M1/M2 balance towards an anti-inflammatory profile (66). In another mouse study involving hepatic ischemia–reperfusion injury, treatment with recombinant VEGF-D facilitated liver repair with expansion of lymphatic vessels and increased expression of R3+ “reparative” macrophages (defined by the authors as Ly6C^low^/CD11b^high^/F4/80^high^/CX3CR1^high^/CCR2^low^). Conversely, VEGFR-3 inhibition suppressed liver repair, drainage function, and accumulation of R3+ reparative macrophages, suggesting that activation of VEGFR-3 signaling induced lymphangiogenesis and increased the number of reparative macrophages essential for liver repair (29).

## R3+ macrophages in disease: foes?

### VEGFR-3+ macrophages signaling in renal fibrosis

Zhang et al. (47) utilized unilateral ureteral obstruction (UUO) and the adriamycin nephropathy mouse model (ARD) to demonstrate the association between renal fibrosis-associated lymphangiogenesis and VEGFR-3-dependent macrophage polarization. VEGF-C-treated macrophages formed tube-like structures in Matrigel *in vitro* and, similarly, adoptive transfusion of primary M1 macrophages into the UUO and ARD mice formed tube-like structures in the fibrotic kidneys. They also identified that the induction of autophagy in macrophages by rapamycin decreased M1 macrophage polarization and differentiation into LECs whereas activation of the VEGF-C/VEGFR-3 pathway downregulated macrophage autophagy and subsequently promoted M1 macrophage polarization. These results suggested that M1 macrophages promoted lymphangiogenesis and contributed to newly formed lymphatic vessels in the renal fibrosis microenvironment, and that VEGF-C/VEGFR3 signaling promoted macrophage M1 polarization by suppressing macrophage autophagy and then increased the transdifferentiation of M1 macrophages into LECs. Another study using db/db mice demonstrated that VEGFR-3 activation increases lymphatic proliferation and M1 macrophage infiltration during tubulointerstitial fibrosis induced by diabetic nephropathy. In this study, palmitate-induced lipotoxicity enhanced M1 phenotypes in RAW264.7 monocytes/macrophages and increased VEGFR-3 and Lyve-1 expression, which were inhibited by SAR131675 (VEGFR-3 inhibitor) treatment, suggesting that inhibiting VEGFR-3 signaling can prevent diabetic nephropathy by inhibiting renal lymphangiogenesis mediated through macrophage polarization under lipotoxic conditions (67).

### VEGFR-3+ tumor-associated macrophages

R3+ macrophages also play a significant role in tumor-associated lymphangiogenesis and cancer cell metastasis. The skin surrounding VEGF-C-transfected melanomas from mice showed a markedly increased density of R3+ macrophages. *In vitro* studies from mouse peritoneal macrophages revealed VEGF-C-induced chemotaxis of macrophages in a dose-dependent manner (33). Macrophages from chemotherapy-treated mice induced lymphangiogenesis in a VEGFR-3-dependent manner by promoting the secretion of cathepsins, leading to the upregulation of VEGF-C by heparanase activity. Blocking VEGFR-3+ tumor-associated macrophages (TAMs) inhibited lymphangiogenesis and subsequent metastasis (48). VEGFR-3+ TAMs increased the VEGF-C/D expression in the bladder tumor mass, resulting in increased lymphatic metastases (39). VEGFC-dependent activation of VEGFR-3 in colorectal TAMs weakened antitumor adaptive immunity promoting cancer immune escape; these effects were abolished upon VEGFR-3 inhibition (68). VEGFR-3-expressing TAMs are also involved in the production of VEGF-C and VEGF-D within the peritumoral stroma, resulting in peritumoral lymphangiogenesis in human cancer (36). Dalton and colleagues identified that R3+ macrophages in the tumor microenvironment orchestrate anti-VEGF therapy resistance. Biopsy specimens from ovarian cancer patients who were resistant to anti-VEGF therapy showed reduced macrophage VEGFR-3 expression. Anti-VEGF antibody (AVA)-resistant immortalized murine macrophages secreted less VEGF and showed increased secretion of alternative pro-angiogenic cytokines and chemokines. These macrophages revealed a significant increase in the methylation at the VEGFR-3 promoter region, suggesting that at the emergence of resistance, depletion of VEGFR-3 expression in macrophages is accompanied by an upregulation of alternative angiogenic pathways, thus facilitating escape from anti-VEGF therapy (69). VEGFR-3 tyrosine kinase activity is also inhibited by VEGFR-targeting and less specific tyrosine kinase inhibitors in use or in development for cancer anti-angiogenic or anti-metastatic effects, such as sunitinib, fruquintinib, or cediranib. Whether reduced vascularization and macrophage numbers, or changes in macrophage polarization, reported with their use are directly through VEGFR-3 activity on macrophages is unclear (26, 70, 71).

### VEGFR-3+ macrophages signaling in obesity

Karaman and colleagues demonstrated that subcutaneous white adipose tissue (SWAT) of high-fat diet (HFD)-fed K14-VEGFR-3-Ig (sR3) mice that constitutively express soluble-VEGFR-3-Ig in the skin, scavenging VEGF-C and -D, had an increased M2/M1 marker expression ratio. A transwell migration experiment revealed VEGFC/D to be highly chemotactic to adipose tissue macrophages particularly M1 macrophages that express heightened VEGFR-3. Antibody mediated depletion of VEGFR-3 in db/db mice reduced macrophage infiltration in adipose tissue, reduced adipose tissue inflammation, and reduced obesity-induced insulin resistance. They suggested that VEGF-C/D plays an important role in recruiting the M1-polarized R3+ macrophages in obese adipose tissue and in the development of obesity-associated insulin resistance (50). Another study from the same group using K14-VEGF-C mice that overexpress human VEGF-C under control of the keratin-14 promoter in epidermal keratinocytes revealed the recruitment of R3+ inflammatory macrophages into SWAT prior to the emergence of weight gain that helped in promoting insulin resistance (51). Overexpression of VEGF-D in adipose tissue of lean mice also drove adipose macrophage infiltration, inflammation, and fibrosis (72).

## VEGFR-3+ macrophages in public databases

In addition to the surface expression or activity confirmed in the studies detailed above and listed in **Table 1**, modern gene array and sequencing data can be examined for macrophage VEGFR-3 expression. We utilized scRNASeqDB to identify *FLT4* expression levels in macrophages from human single-cell transcriptome datasets. This database includes almost all the currently available human single-cell transcriptome datasets (*n* = 38) covering 200 human cell lines or cell types and 13,440 samples (https://bioinfo.uth.edu/scrnaseqdb/). Within the macrophages across all datasets in the single-cell RNA sequencing database, the expression of *FLT4* is ranked in the top 45%, depicting that it is moderately expressed in macrophages compared to other genes. *FLT4* expression in macrophages was demonstrated with a maximum transcripts per million (TPM) value of 8.6 (73). The National Library of Medicine’s Gene Expression Omnibus (https://www.ncbi.nlm.nih.gov/geoprofiles) has an array of studies demonstrating even higher expression levels when searched for “flt4 macrophage” or “flt4 monocyte”. However, despite the abundant findings of R3+ macrophages or VEGFR-3 responsiveness of macrophages in the literature, they are largely lacking from recent single-cell RNA-seq data. This scarcity may potentially be attributed to their categorization as endothelial cells during the analytical process (*FLT4* being highest in lymphatic or some blood endothelium) or transient expression during disease or monocyte differentiation as discussed above.

## Conclusions

**Figure 1:**
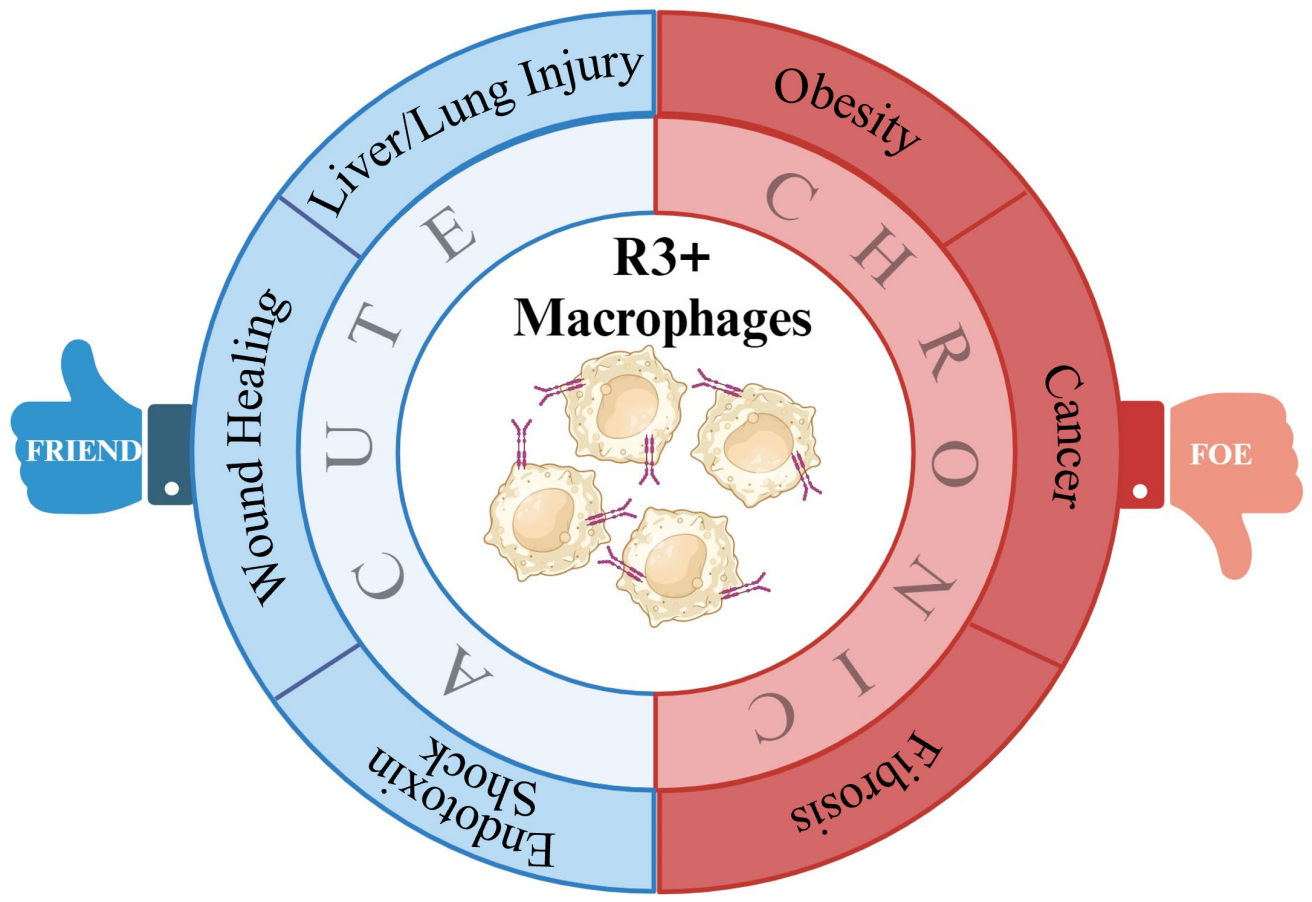
Summary of potential VEGFR-3+ macrophage roles in disease. By impacting macrophage polarization, VEGFR-3 signaling in macrophage can make them either friends or foes to inflammatory resolution in various pathologies.

VEGF-C/VEGFR-3 signaling has been identified to regulate macrophage polarization. In acute hepatic injury, VEGFR-3 signaling modified the polarization of macrophages from M1 to M2. Macrophages isolated from colitic mice demonstrated M2 characteristics when treated with VEGF-C. In obesity, VEGFR-3 expression is upregulated in M1 macrophages and VEGFR-3 signal inhibition significantly increase M2 macrophage numbers. Similarly, in renal fibrosis, VEGF-C/VEGFR-3 signaling promotes M1 polarization and increases the potential transdifferentiation of M1 macrophages into LECs. R3+ macrophage signaling is beneficial in certain pathological conditions including liver/lung injury, wound healing, infection, and inflammatory disorders where tissue-specific induction of VEGFR-3 signaling might be of therapeutic benefit. In contrast to these settings, diseases including obesity, cancer, and fibrosis are characterized by elevated VEGF-C and VEGF-D levels and increased VEGFR-3 signaling. In these cases, inhibition of signaling via this axis, either by VEGFC/D traps or by VEGFR-3 small-molecule inhibitors, stands to be of therapeutic benefit (**Figure 1**).

Lymphatic responses and macrophage polarization varies among disease conditions. Based on the current literature, it is suggested that M1 macrophages drive the inflammatory response after an injury triggering innate immunity and promoting lymphangiogenesis, which might assist in immune clearance, while in a chronic inflammatory environment, pro-inflammatory M1 macrophages persist without transitioning to anti-inflammatory M2 phenotypes contributing to impairment in tissue repair. Improving our understanding of the basic mechanisms underlying macrophage plasticity and the impact of lymphangiogenesis and lymphatic–immune signaling on acute and chronic pathological conditions will help determine if R3+ macrophages are a potential therapeutic or diagnostic target for various diseases in the future when VEGFR-3 or pan-tyrosine kinase signaling is manipulated.

## Author contributions

SK: Conceptualization, Data curation, Investigation, Writing – original draft, Writing – review & editing. JR: Conceptualization, Funding acquisition, Supervision, Writing – review & editing.
